# Predictive value of clinical and laboratory features for the main febrile diseases in children living in Tanzania: A prospective observational study

**DOI:** 10.1371/journal.pone.0173314

**Published:** 2017-05-02

**Authors:** Olga De Santis, Mary Kilowoko, Esther Kyungu, Willy Sangu, Pascal Cherpillod, Laurent Kaiser, Blaise Genton, Valérie D’Acremont

**Affiliations:** 1 Department of Ambulatory Care and Community Medicine, University of Lausanne, Lausanne, Switzerland; 2 University of Barcelona, Barcelona, Spain; 3 Amana Hospital, Dar es Salaam, United Republic of Tanzania; 4 St-Francis Hospital, Ifakara, United Republic of Tanzania; 5 Ilala Municipal Council, Dar es Salaam, United Republic of Tanzania; 6 Laboratory of Virology, Division of Infectious Diseases, University Hospital of Geneva, and Faculty of Medicine, University of Geneva, Geneva, Switzerland; 7 Infectious Diseases Service, Lausanne University Hospital, Lausanne, Switzerland; 8 Swiss Tropical and Public Health Institute, University of Basel, Basel, Switzerland; Liverpool School of Tropical Medicine, UNITED KINGDOM

## Abstract

**Background:**

To construct evidence-based guidelines for management of febrile illness, it is essential to identify clinical predictors for the main causes of fever, either to diagnose the disease when no laboratory test is available or to better target testing when a test is available. The objective was to investigate clinical predictors of several diseases in a cohort of febrile children attending outpatient clinics in Tanzania, whose diagnoses have been established after extensive clinical and laboratory workup.

**Method:**

From April to December 2008, 1005 consecutive children aged 2 months to 10 years with temperature ≥38°C attending two outpatient clinics in Dar es Salaam were included. Demographic characteristics, symptoms and signs, comorbidities, full blood count and liver enzyme level were investigated by bi- and multi-variate analyses (Chan, et al., 2008). To evaluate accuracy of combined predictors to construct algorithms, classification and regression tree (CART) analyses were also performed.

**Results:**

62 variables were studied. Between 4 and 15 significant predictors to rule in (aLR+>1) or rule out (aLR+<1) the disease were found in the multivariate analysis for the 7 more frequent outcomes. For malaria, the strongest predictor was temperature ≥40°C (aLR+8.4, 95%CI 4.7–15), for typhoid abdominal tenderness (5.9,2.5–11), for urinary tract infection (UTI) age ≥3 years (0.20,0–0.50), for radiological pneumonia abnormal chest auscultation (4.3,2.8–6.1), for acute HHV6 infection dehydration (0.18,0–0.75), for bacterial disease (any type) chest indrawing (19,8.2–60) and for viral disease (any type) jaundice (0.28,0.16–0.41). Other clinically relevant and easy to assess predictors were also found: malaria could be ruled in by recent travel, typhoid by jaundice, radiological pneumonia by very fast breathing and UTI by fever duration of ≥4 days.

The CART model for malaria included temperature, travel, jaundice and hepatomegaly (sensitivity 80%, specificity 64%); typhoid: age ≥2 years, jaundice, abdominal tenderness and adenopathy (46%,93%); UTI: age <2 years, temperature ≥40°C, low weight and pale nails (20%,96%); radiological pneumonia: very fast breathing, chest indrawing and leukocytosis (38%,97%); acute HHV6 infection: less than 2 years old, (no) dehydration, (no) jaundice and (no) rash (86%,51%); bacterial disease: chest indrawing, chronic condition, temperature ≥39.7°c and fever duration >3 days (45%,83%); viral disease: runny nose, cough and age <2 years (68%,76%).

**Conclusion:**

A better understanding of the relative performance of these predictors might be of great help for clinicians to be able to better decide when to test, treat, refer or simply observe a sick child, in order to decrease morbidity and mortality, but also to avoid unnecessary antimicrobial prescription. These predictors have been used to construct a new algorithm for the management of childhood illnesses called ALMANACH.

## Introduction

For many years, acute febrile illnesses have been presumptively treated as malaria cases in Sub-Saharan Africa. The epidemiological context has now changed with malaria transmission declining in many areas of the continent [2] and thus most febrile episodes being due to other causes than malaria. Infectious diseases still account for the vast majority of child deaths in developing countries, the main non-malaria causes being pneumonia and diarrhea [3]. Therefore, appropriate management of febrile illnesses is a priority which requires an integrated approach and continuous research efforts [4] to improve existing guidelines.

A recent study has looked at the causes of fever in young children in Tanzania and found that more than half had an acute respiratory infection (ARI). However, only few had a radiologically confirmed pneumonia and a virus was found in 4 out of 5 of these children. Besides malaria, most nonspecific fevers were also due to cosmopolitan viruses, such as Human Herpes Virus 6 (HHV6) and parvovirus B19, while bacterial diseases, such as urinary tract infection (UTI), typhoid fever, occult bacteremia or rickettsiosis were less frequent [5]. In resource-limited countries, medical equipment is scarce and most laboratory tests used for the diagnosis of infectious diseases are not available or too expensive. In such a situation, a way to improve fever management is to look for predictors, namely demographic characteristics, specific exposures or clinical symptoms or signs that can be obtained from the patient relatively easily. Several studies have investigated the performance of clinical predictors of specific childhood illnesses. Some were conducted in emergency departments of developed countries, usually to identify serious bacterial diseases [6–10]. Others, conducted in developing countries, have focused on one disease only, such as malaria [11–13], typhoid fever [14–17], urinary tract infection [13,18], pneumonia [19,20], bacterial gastroenteritis [21,22], bacteremia [23] and dengue [24]. To our knowledge, there is no study that has assessed the predictive value of clinical and laboratory predictors of the different causes of fever simultaneously in the same group of patients. This latter approach would allow to produce the most accurate results, avoiding bias due to patient selection and misclassification of fever causes. The objective of the present study was to investigate predictors of the seven most frequent causes of fever in children included consecutively in the above-mentioned Tanzanian study [5]. The aim was to generate evidence-based information, presented in a user-friendly format to offer clinicians an updated view of the performance of symptoms and signs as predictors of the most common febrile diseases. These results could also be used to develop or update guidelines for the management of febrile children at the outpatient level.

## Methods

### Study design and setting

The study was based on case-control analyses within a prospective study aimed at establishing the precise cause of the fever, which was conducted from April to December 2008 in two outpatient clinics in Tanzania: one in Dar-es-Salaam, the economic capital (urban site) and one in Ifakara, Morogoro region, situated in the center of the country (rural site).

### Study population, inclusion and exclusion criteria

The methodology of the study is detailed elsewhere [5]. In brief, 1005 consecutive children aged 2 months to 10 years (94% were less than five years) presenting with an axillary temperature of ≥38°C were screened. Two additional inclusion criteria were used: 1) visit was the first for the present illness; 2) fever duration ≤1 week. Exclusion criteria were: 1) chief complaint was injury or trauma; 2) receipt of antimalarials or antibiotics in the preceding week; 3) severe malnutrition according to WHO definition [25]; 4) requirement of immediate life-saving procedures. To record clinical findings, all items relevant to a febrile presentation were listed in a standardized case report form. In all cases, blood samples (5ml whenever possible), pooled nasal and throat swabs were taken for microbiologic testing on-site (rapid tests and blood cultures) and further serologic and molecular work-up. A systematic set of investigations was performed in all patients while additional investigations (including chest X-ray) were carried out according to several non-mutually exclusive decision charts developed for chief complaint(s). The most appropriate microbiologic method of detection was selected for the diagnosis of each pathogen in an acute episode.

The details of the study were discussed with the children’s guardians who provided written informed consent. The protocol was approved by the National Institute for Medical Research Review Board in Tanzania and the local ethical committee in Basel, Switzerland.

### Predictors’ definitions

The parameters were divided in four groups: 1) symptoms based on history taking; 2) signs based on clinical examination; 3) epidemiological and demographic parameters (age, rural/urban site, season, chronicity, recent travel); and 4) laboratory test results. All parameters were included in the statistical analysis for each outcome, except if they were part of the criteria used to define this outcome (Table 1) or if present in less than 10 children. Laboratory parameters measured in a selected subgroup of patients were not included in the analyses (eosinophils, creatinine, C-reactive protein (CRP), procalcitonin (PCT) and other host biomarkers) and, for pneumonia, are the subject of another publication [26]. When two predictors were similar from the clinical or pathophysiological point of view [for example erythropenia and anemia (based on hemoglobin)], and thus strongly correlated, only the easiest parameter to assess or measure (in this case anemia) was retained for the multivariate analysis.

**Table 1:** Pre-defined case definitions. Pre-defined case definitions based on clinical and laboratory criteria, for each febrile illness included in the present analysis.

	Clinical presentations considered for the diagnosis	Basic criteria needed for a documented diagnosis (usually clinical)	Additional criteria needed for a documented diagnosis (usually laboratory test or X-ray)	Parameters not included in the analysis because part of the case definition
**Malaria**	All presentations	None	Positive malaria RDT or blood smear	
**Typhoid**	All presentations	None	Positive typhoid RDT OR blood or stool culture positive for *Salmonella enterica serovar Typhi*	
**Urinary tract infection**	Presentations without a clear diagnosis after clinical examination ^α^	Leukocytes or nitrites on urine dipstick	Positive urine culture (non-contaminant bacteria ≥ 10^4^ cfu/ml, other than mixed growth)	Fast breathing, chest indrawing, abnormal findings on chest auscultation, diarrhea^Ω^
**Radiological pneumonia**	Presentations with cough	Fast breathing OR chest indrawing OR abnormal findings on chest auscultation	Primary end-point consolidation ^π^ on chest radiography	Cough, fast breathing ^β^
**HHV6 infection**	All presentations	None	Positive PCR for human herpesvirus 6 (>1000 copies/ml)	

RDT: Rapid Diagnostic Test; cfu: colony forming units; HHV6: Human Herpes Virus 6; PCR: Polymerase Chain Reaction

^α^Clear clinical diagnosis: meningitis, chickenpox, tonsillitis, fast breathing, chest indrawing, abnormal findings

^β^ “chest indrawing” and “abnormal findings on chest auscultation” could still be studied as almost all patients were diagnosed with clinical pneumonia on the basis of “fast breathing”

^π^ according to the WHO’s Pneumococcal Trialist Ad Hoc Committee recommendations (ref: Cherian T et al, Bull World Health Organ 2005; 83:353–9).

^Ω ^signs used as criteria for other diagnoses in the initial algorithm of the Tanzanian fever study (see D’Acremont V. et al, 2014, NEJM, suppl. Fig 1 (5), excluding the possibility of UTI diagnosis.

### Diseases’ definitions

The criteria and diagnostic procedures to define each disease causing fever have been described in a previous publication [5]. Briefly, the final diagnoses were established based on pre-defined criteria derived from WHO and Infectious Diseases Society of America guidelines, as well as systematic reviews. In order to maximize consistency and to minimize subjectivity, these were generated by means of a computer-based algorithm. Diseases that were documented by at least one microbiological or radiological test and affecting at least 30 patients were selected for the analyses. They included diseases documented with laboratory tests performed in all children (malaria, typhoid fever, acute HHV6), or diseases documented on one laboratory or radiological test combined with a constellation of clinical parameters (UTI, radiological pneumonia). Diseases were not mutually exclusive and some patients had more than one diagnosis. The pre-defined case definitions for each disease included in the analysis, as well as the parameters excluded from the analysis because they were part of this definition, are presented in Table 1.

An analysis was also performed for viral disease that included all clinically or microbiologically confirmed viral diagnoses, i.e. acute respiratory infection with one or more positive nasopharyngeal PCR results for a respiratory virus, gastroenteritis with a positive rapid diagnostic test result for adenovirus or rotavirus, systemic viral infections (acute HHV6, parvovirus B19, EBV, CMV and hepatitis A) documented by PCR or serology, and nasopharyngeal viral infections (defined as fever with no documented cause except a positive nasopharyngeal PCR result for a respiratory virus). ‘Bacterial diseases’ included all clinically or microbiologically confirmed bacterial diagnoses, i.e.: pneumonia with an end-point consolidation on chest X-ray (see Table 1) or severe acute respiratory infections, with a positive nasopharyngeal PCR result for *Streptococcus pneumoniae*; typhoid fever; UTI; sepsis due to bacteremia; gastroenteritis with a positive stool culture result for *Salmonella* or *Shigella*; rickettsioses; Q fever; leptospiroses and significant skin and mucosal purulent infections (see the supplementary appendix of the Tanzanian Fever study publication for more details [5].

### Statistical analysis

Data were analyzed using STATA version 12 (College Station Texas, USA) and R software (version 2.15.3). To calculate the crude likelihood ratios (LR) for the bivariate analysis, the qualitative parameters were coded as dichotomous and the continuous parameters were dichotomized using accepted thresholds for normality. Multivariate analyses, using the Spiegelhalter-Knill-Jones (SKJ) approach [1] to obtain adjusted likelihood ratios (aLR), were performed. Only clinical parameters with a crude LR+ >2 or LR- <0.5 were introduced in the stepwise backward logistic regression models. Parameters were then excluded from the model if the p-value was ≥0.05 according to the Wald test. Confidence intervals were calculated using a bootstrap method.

To reduce the statistical hazard, parameters were also tested using a different type of statistical analysis: the classification and regression tree (CART). CART analyses have the added benefit to evaluate the combined accuracy of predictors to correctly predict the outcomes. The aim of performing this second analysis was not to provide ready-to-use clinical guidelines but to identify the best combinations of predictors. The same clinical parameters as for the SKJ approach (crude LR+ >2 or LR- <0.5) were introduced in the formula to develop the CART. Continuous predictors were both introduced as such in the CART models to let the software decide the best cutoff and were introduced as dichotomous variables with usual cutoffs. A minimum of 10 cases for parent nodes and 5 cases for child nodes, and a maximum tree depth of 4 nodes were used as conditions to generate the decision trees. The cost of misclassifying the presence versus the absence of an outcome was systematically varied to generate models favoring higher sensitivity or specificity. The pruning was varied to avoid overfitting. CART models were compared based on sensitivity, specificity and LRs. Analyses were performed using the ‘mass’, ‘boot’, ‘rpart’, ‘caret’ and ‘rpart.plot:prp’ libraries in R software.

## Results

One thousand and ten (510 in Dar es Salaam and 500 in Ifakara) consecutive febrile children were enrolled and 5 subsequently excluded (consent withdrawn [2]; failure of blood withdrawal [1], inclusion criteria not met [2]). Median age was 18.2 months, 47% were females, 86% had a body weight within 3 standard deviations of the population mean, the median axillary temperature was 38.4°C (range 38.0 to 41.2°C) and fever was the chief main complaint cited by the guardian in 66.4% of the patients. 62 potential predictors of 5 different febrile diseases and 2 groups of diseases (viral/bacterial) could be analyzed. The analyses were based on 105 cases of malaria, 37 cases of typhoid fever, 59 cases of UTI, 31 cases of radiological pneumonia, 79 cases of acute HHV6 infection, 221 cases of bacterial disease and 708 cases of viral disease. 21 (2%) children had more than one diagnosis (4 malaria+typhoid, 1 malaria+pneumonia, 2 malaria+UTI, 2 malaria+HHV6, 4 typhoid+pneumonia, 1 typhoid+UTI, 1 typhoid+HHV6, 1 pneumonia+HHV6 and 5 UTI+HHV6). Out of the 62 variables studied, 16 (see S1 Table) were not predictive of any of the diseases including someone sick in the environment, rural versus urban site, vomiting, abdominal pain, cough duration of ≤ 3 days, cough duration of > 3 days. Results of the bivariate analyses are presented in Table 2. Results of the multivariate analyses are presented in Table 3, as adjusted LR (aLR).

**Table 2:** Crude likelihood ratios (LR) for the predictors of each febrile illness (bivariate analysis). Only results for which the LR+ was 2 and above or the LR- was 0.5 and below are provided (otherwise the cell is left empty; if not applicable, NA is mentioned). LR with a 95% confidence interval (CI) that did not include 1 are in bold.

Predictorsn	n	Malaria (105 cases)				Typhoid Fever (37 cases)				Urinary tract infection (59 cases)				Radiological pneumonia (31 cases)				Acute HHV6 infection (79 cases)				Bacterial disease (221 cases)				Viral disease (708 cases)			
		LR+ (95%CI)		LR- (95%CI)		LR+ (95%CI)		LR- (95%CI)		LR+ (95%CI)		LR- (95%CI)		LR+ (95%CI)		LR- (95%CI)		LR+ (95%CI)		LR- (95%CI)		LR+ (95%CI)		LR- (95%CI)		LR+ (95%CI)		LR- (95%CI)	
Age < 2 years	616									**1,4**	**(1,2-1,6)**	**0,42**	**(0,24-0,75)**					**1,5**	**(1,4-1,7)**	**0,25**	**(0,13-0,48)**								
Age < 3 years	781									1,2	(1,2-1,3)	0,22	(0,072-0,66)					1,21	(1,1-1,3)	0,32	(0,15-0,70)								
Age < 5 years	941																	1,0	**(1,0-1,1)**	0,38	**(0,094-1,5)**					**1,07**	**(1,0-1,1)**	**0,42**	**(0,26-0,67)**
Age ≥ 5 years	64	**2,6**	**(1,5-4,5)**	**0,91**	**(0,84-0,98)**	**3,2**	**(1,6-6,6)**	0,86	(0,74-1,0)																				
Recent travel	49	**2,8**	**(1,5-5,1)**	**0,92**	**(0,86-0,99)**													0,50	(0,12-2,0)	1,0	(0,99-1,1)	0,50	(0,21-1,1)	1,0	(1,0-1,1)				
Chronic condition	70													2,4	(0,92-6,1)	0,92	(0,80-1,1)					**3,0**	**(1,8-4,9)**	**0,92**	**(0,87-0,97)**				
Rainy season (vs dry season)	576					**1,4**	**(1,2-1,7)**	**0,50**	**(0,27-0,92)**																				
Difficulty to drink	12					2,4	(0,32-18)	0,98	(0,93-1,0)									2,3	(0,52-11)	0,99	(0,95-1,0)								
Difficulty to eat	40																									**0,38**	**(0,21-0,70)**	**1,05**	**(1,0-1,1)**
Fever duration of ≤ 3 days	888																									**1,13**	**(1,1-1,2)**	**0,46**	**(0,33-0,64)**
Fever duration of > 3 days	97					**2,1**	**(1,0-4,1)**	0,89	(0,76-1,0)	**2,0**	**(1,2-3,6)**	0,89	(0,79-1,0)									**2,1**	**(1,4-3,0)**	**0,91**	**(0,85-0,97)**	**0,46**	**(0,31-0,66)**	**1,10**	**(1,0-1,2)**
Seizure	16	**3,9**	**(1,4-11)**	0,96	(0,92-1,0)									2,1	(0,29-15)	0,98	(0,92-1,0)					**4,6**	**(1,7-12)**	0,97	(0,94-1,0)	**0,33**	**(0,12-0,87)**	1,02	(1,0-1,0)
Headache	61	**2,6**	**(1,5-4,5)**	**0,91**	**(0,85-0,99)**																								
Runny nose	464																									**2,88**	**(2,3-3,7)**	**0,53**	**(0,48-0,59)**
Cough (any duration)	458													NA	NA	NA	NA									**2,45**	**(2,0-3,1)**	**0,58**	**(0,52-0,64)**
Dyspnea	20	0,45	(0,061-3,3)	1,0	(0,99-1,0)									**7,9**	(2,8-22)	0,89	(0,77-1,0)					**4,3**	**(1,8-10)**	**0,96**	**(0,93-0,99)**				
≥ 4 liquid stool/day	45																									**0,44**	**(0,25-0,77)**	**1,04**	**(1,0-1,1)**
Dehydration	83					0,32	(0,050-2,2)	1,06	(1,0-1,1)					2,0	(0,88-4,6)	0,91	(0,78-1,1)	0,29	(0,070-1,2)	1,07	(1,0-1,1)								
Pollakiuria	28	0,32	(0,044-2,3)	**1,0**	**(1,0-1,0)**					**3,5**	**(1,4-8,8)**	0,94	(0,87-1,0)	2,4	(0,60-9,7)	0,96	(0,88-1,1)												
Rash	38	**2,3**	**(1,1-4,9)**	0,96	(0,90-1,0)																								
Low weight	29									2,6	(0,93-7,2)	0,96	(0,89-1,0)	2,3	(0,57-9,3)	0,96	(0,88-1,1)					**5,0**	**(2,4-10)**	**0,94**	**(0,90-0,97)**	**0,45**	**(0,22-0,92)**	1,03	(1,0-1,1)
Temperature ≥ 39°C	225	**2,4**	**(1,9-3,1)**	**0,65**	**(0,54-0,78)**									**2,1**	(1,4-3,1)	**0,70**	(0,51-0,97)												
Temperature ≥ 40°C	37	**10**	**(5,5-19)**	**0,83**	**(0,75-0,91)**	**3,2**	**(1,2-8,5)**	0,92	(0,82-1,0)	**3,1**	**(1,3-7,1)**	0,93	(0,85-1,0)					0,33	(0,045-2,3)	1,0	(1,0-1,1)	**2,2**	**(1,1-4,1)**	0,96	(0,93-1,0)	**0,32**	**(0,17-0,60)**	**1,05**	**(1,0-1,1)**
Tachycardiac														**3,5**	(2,0-6,1)	**0,75**	(0,58-0,95)												
Fast breathingd	244									NA	NA	**1,3**	**(1,2-1,4)**	NA	NA	NA	NA									**2,21**	**(1,6-3,0)**	**0,82**	**(0,77-0,87)**
Very fast breathinge	68													**6,1**	(3,5-10)	**0,69**	(0,53-0,89)					**2,3**	**(1,5-3,7)**	**0,93**	**(0,88-0,98)**	**2,75**	**(1,4-5,5)**	**0,95**	**(0,92-0,97)**
Chest indrawing	27	0,33	(0,045-2,4)	1,0	(1,0-1,0)					NA	NA			**7,1**	(2,9-18)	0,86	(0,74-1,0)					**28**	**(8,6-93)**	**0,89**	**(0,85-0,94)**	**5,24**	**(1,2-22)**	**0,97**	**(0,95-0,99)**
Nasal flaring	22													**7,0**	(2,5-19)	0,89	(0,77-1,0)					**35**	**(8,4-150)**	**0,91**	**(0,87-0,95)**	4,19	(0,99-18)	**0,98**	**(0,96-0,99)**
Abnormal chest auscultation	108	0,50	(0,23-1,1)	**1,1**	**(1,0-1,1)**					NA	NA			**5,5**	(3,7-8,1)	**0,53**	(0,37-0,77)									**2,60**	**(1,5-4,4)**	**0,91**	**(0,88-0,95)**
Jaundice	42	**7,8**	**(4,4-14)**	**0,83**	**(0,76-0,91)**	**6,2**	**(3,1-12)**	**0,81**	**(0,69-0,96)**													**2,9**	**(1,6-5,3)**	**0,94**	**(0,90-0,98)**	**0,11**	**(0,055-0,24)**	**1,11**	**(1,1-1,2)**
Pale nails	25	**4,0**	**(1,8-9,1)**	0,94	(0,89-1,0)	**3,6**	**(1,1-11)**	0,94	(0,85-1,0)	**3,1**	**(1,1-8,6)**	0,95	(0,89-1,0)									**2,8**	**(1,3-6,1)**	0,97	(0,94-1,0)	**0,33**	**(0,15-0,72)**	**1,03**	**(1,0-1,1)**
White mouth	13									**4,8**	**(1,4-17)**	0,96	(0,90-1,0)	2,6	(0,35-20)	0,98	(0,92-1,0)	2,3	(0,52-11)	0,99	(0,95-1,0)	**3,0**	**(1,0-9,0)**	0,98	(0,96-1,0)	0,36	(0,12-1,1)	1,02	(1,0-1,0)
Mouth ulcer	39					**3,0**	**(1,1-8,0)**	0,93	(0,83-1,0)																				
Lymphadenopathy	48	0,18	(0,025-1,3)	**1,0**	**(1,0-1,1)**	**3,0**	**(1,3-7,2)**	0,91	(0,80-1,0)					**2,9**	(1,1-7,5)	0,91	(0,80-1,0)					**3,0**	**(1,7-5,2)**	**0,93**	**(0,89-0,97)**				
Abdominal tenderness	33	**2,3**	**(1,0-5,2)**	0,96	(0,91-1,0)	**7,0**	**(3,3-15)**	**0,83**	**(0,71-0,97)**					**4,3**	**(1,6-12)**	0,90	(0,78-1,0)												
Hepatomegaly	45	**3,5**	**(1,9-6,4)**	**0,91**	**(0,84-0,98)**	**4,0**	**(1,8-8,9)**	0,87	(0,76-1,0)	2,0	(0,82-4,9)	0,96	(0,88-1,0)									**2,8**	**(1,6-5,0)**	**0,94**	**(0,90-0,98)**	**0,34**	**(0,19-0,59)**	**1,06**	**(1,0-1,1)**
Liver pain	11	3,2	(0,87-12)	0,98	(0,95-1,0)	**9,8**	**(2,7-35)**	0,93	(0,84-1,0)	3,6	(0,79-16)	0,98	(0,93-1,0)	3,1	(0,41-24)	0,98	(0,92-1,0)					**4,3**	**(1,3-14)**	0,98	(0,96-1,0)	**0,09**	**(0,020-0,43)**	**1,03**	**(1,0-1,0)**
Splenomegaly	44	**6,5**	**(3,7-11)**	**0,84**	**(0,77-0,92)**	**4,1**	**(1,9-9,2)**	0,87	(0,76-1,0)													**3,0**	**(1,7-5,3)**	**0,94**	**(0,90-0,98)**	**0,20**	**(0,11-0,36)**	**1,09**	**(1,0-1,1)**
Anemia (Hb < 8 g/dl)	138	**2,6**	**(1,8-3,6)**	**0,79**	**(0,69-0,90)**																								
Severe anemia (Hb < 5 g/dl)	14	2,3	(0,66-8,2)	0,98	(0,95-1,0)	**4,4**	**(1,0-19)**	0,96	(0,89-1,0)													**8,9**	**(2,8-28)**	**0,96**	**(0,93-0,99)**				
Thrombcytopeniaf	181	**3,0**	**(2,3-3,9)**	**0,65**	**(0,55-0,77)**									0,36	(0,094-1,4)	**1,1**	**(1,0-1,3)**												
Leukocytosisg	254													**2,6**	**(2,0-3,5)**	**0,48**	(0,30-0,77)												
Leucopeniah	80	**3,3**	**(2,1-5,1)**	**0,84**	**(0,76-0,93)**					0,20	(0,029-1,4)	**1,1**	**(1,0-1,1)**					**3,4**	**(2,1-5,4)**	**0,83**	**(0,73-0,93)**								
Lymphocytosisi	124									**2,0**	**(1,2-3,3)**	0,86	(0,75-1,0)																
Lymphopeniaj	175													0,37	(0,097-1,4)	**1,1**	**(1,0-1,3)**	**2,4**	**(1,8-3,3)**	**0,74**	**(0,62-0,88)**								
High ALTk	43					**3,4**	**(1,4-8,2)**	0,90	(0,79-1,0)									0,28	(0,039-2,0)	1,0	(1,0-1,1)								

c Heart rate > 180 if age < 24 months; heart rate > 140 if age ≥ 24 and < 72 months; heart rate > 130 if age ≥ 72 months.

d Respiratory rate ≥ 50 if age < 12 months; respiratory rate ≥ 40 if age ≥ 12 months.

e Respiratory rate ≥ 60 if age < 12 months; respiratory rate ≥ 50 if age ≥ 12 months.

f Platelets < 150 G/l.

g White Blood Cells > 17.5 G/l if age < 24 months; WBC > 15.5 G/l if age ≥ 24 and < 72 months; WBC > 13.5 G/l if age ≥ 72 months.

h WBC < 5 G/l if age < 24 months; WBC < 6 G/l if age ≥ 24 and < 72 months; WBC < 4.5 G/l if age ≥ 72 months.

i Lymphocytes > 9 G/l if age ≤ 12 months; lymphocytes > 7 G/l if age > 12 months.

j Lymphocytes < 3 G/l if age ≤ 24 months; lymphocytes < 1.5 G/l if age > 24 months.

k Alanine aminotransferase > 62 UI/l.

n in bold the LR with CI not including the 1.

**Table 3:** Adjusted likelihood ratios (LR) for the predictors of each febrile illness (multivariate analysis). Results with a 95% CI that did not include 1 are in bold.

Malaria n = 105					
**Predictors variables**	**n**	**aLR+**	**(95% CI)**	**aLR-**	**(95% CI)**
**Temperature ≥40°C**	37	**8.4**	**(4.7-15)**	**0.84**	**(0.76-0.91)**
**Splenomegaly**	44	**5.0**	**(2.9-8.1)**	**0.86**	**(0.80-0.93)**
Seizures	16	4.1	(0.84.11)	0.96	(0.92-1)
Rash	38	4.0	(0.75-14)	0.93	(0.83-1)
Bulging tympanum	11	3.9	(0-16)	0.98	(0.93-1)
**Recent travel**	49	**3.5**	**(1.4-7.1)**	**0.91**	**(0.82-0.98)**
**Age ≥ 5 years**	64	**3.3**	**(1.6-6.1)**	**0.88**	**(0.79-0.97)**
**Jaundice**	42	**3.3**	**(2.3-4.6)**	**0.90**	**(0.84-0.95)**
**Thrombocytopenia**	181	**2.9**	**(2.1-3.7)**	**0.66**	**(0.54-0.77)**
**Leucopenia**	80	**2.6**	**(1.7-3.6)**	**0.87**	**(0.79-0.94)**
**Headache**	61	**2.5**	**(1.3-4.2)**	**0.92**	**(0.85-0.98)**
**Anemia**	138	**2.2**	**(1.6-2.9)**	**0.82**	**(0.73-0.90)**
Pollakiuria	28	0.23	(0-1.2)	1.03	(0.99-1.1)
Liver pain	11	0.11	(0.01-1.4)	1.04	(0.99-1.1)
**Lymphadenopathy**	48	**0.07**	**(0-0.5)**	**1.07**	**(1-1.1)**
Typhoid fever n = 37					
**Predictors variables**	**n**	**aLR+**	**(95% CI)**	**aLR-**	**(95% CI)**
**Abdominal tenderness**	33	**5,9**	**(2,5-11)**	**0,84**	**(0,71-0,96)**
Liver pain	11	3,9	(0-8,4)	0,95	(0,89-1,0)
Lymphadenopathy	48	3,1	(0,68-6,1)	0,90	(0,78-1,0)
High alanine aminotransferase	43	3,0	(0,86-5,5)	0,91	(0,80-1,0)
Mouth ulcer	39	2,9	(0,62-6,7)	0,92	(0,81-1,0)
Temperature ≥40°C	37	2,7	(0,80-5,1)	0,93	(0,83-1,0)
**Jaundice**	42	**2,5**	**(1,6-3,6)**	**0,90**	**(0,80-0,97)**
**Rainy season (vs dry season)**	576	**1,3**	**(1,1-1,5)**	**0,56**	**(0,25-0,89)**
Urinary tract infection n = 59					
**Predictors variables**	**n**	**aLR+**	**(95% CI)**	**aLR-**	**(95% CI)**
Pollakiuria	28	5,4	(0,70-16)	0,92	(0,82-1,0)
White mouth	13	4,6	(0-14)	0,96	(0,90-1,0)
**Temperature ≥40°C**	37	**4,1**	**(1,0-9,6)**	**0,91**	**(0,81-1,0)**
Low weight	29	3,4	(0,42-11)	0,94	(0,85-1,0)
Pale nails	25	3,2	(0,51-7,9)	0,95	(0,87-1,0)
**Fever duration >3 days**	97	**1,9**	**(1,1-2,9)**	**0,91**	**(0,80-1,0)**
**Age <3 years**	781	**1,3**	**(1,2-1,3)**	**0,20**	**(0-0,50)**
**Leucopenia**	80	**0,20**	**(0-0,70)**	**1,1**	**(1,0-1,1)**
Radiological pneumonia n = 31					
**Predictors variables**	**n**	**aLR+**	**(95% CI)**	**aLR-**	**(95% CI)**
Abdominal tenderness	33	5,7	(1,0-15)	0,88	(0,73-1,0)
**Abnormal chest auscultation**	108	**4,3**	**(2,8-6,1)**	**0,59**	**(0,39-0,78)**
Pollakiuria	28	3,7	(0-15)	0,94	(0,81-1,0)
**Very fast breathing**	68	**3,6**	**(2,3-5,1)**	**0,76**	**(0,62-0,89)**
Chronic condition	70	2,7	(0,50-6,2)	0,91	(0,75-1,0)
**Leucocytosis**	254	**2,2**	**(1,5-2,7)**	**0,55**	**(0,33-0,76)**
**Temperature ≥ 39°C**	225	**1,9**	**(1,2-2,7)**	**0,74**	**(0,51-0,96)**
**Thrombocytopenia**	181	**0,37**	**(0-0,98)**	**1,1**	**(1,0-1,2)**
HHV6 infection n = 79					
**Predictors variables**	**n**	**aLR+**	**(95% CI)**	**aLR-**	**(95% CI)**
**Leucopenia**	80	**3,1**	**(1,9-4,7)**	**0,84**	**(0,74-0,93)**
**Lymphopenia**	175	**1,9**	**(1,5-2,4)**	**0,79**	**(0,70-0,90)**
**Age <2 years**	616	**1,6**	**(1,4-1,8)**	**0,22**	**(0,081-0,39)**
Dehydration	83	0,18	(0-0,75)	1,1	(1,0-1,1)
Bacterial disease n = 221					
**Predictors variables**	**n**	**aLR+**	**(95% CI)**	**aLR-**	**(95% CI)**
**Chest indrawing**	27	**19**	**(8,2-60)**	**0,90**	**(0,86-0,94)**
**Nasal flaring**	22	**11**	**(5,4-22)**	**0,94**	**(0,91-0,96)**
**Severe anemia**	14	**9,7**	**(2,8-48)**	**0,96**	**(0,93-0,98)**
**Seizures**	16	**5,9**	**(1,5-26)**	**0,96**	**(0,92-0,99)**
**Low weight**	29	**4,6**	**(2,2-9,4)**	**0,94**	**(0,90-0,97)**
**Lymphadenopathy**	48	**3,5**	**(1,9-6,3)**	**0,92**	**(0,88-0,97)**
White mouth	13	3,4	(0,73-15)	0,98	(0,95-1,0)
Temperature>40°C	37	2,4	(0,95-5,5)	0,96	(0,92-1,0)
**Jaundice**	42	**2,0**	**(1,3-3,0)**	**0,96**	**(0,93-0,99)**
**Fever duration >3 days**	97	**1,6**	**(1,3-2,1)**	**0,94**	**(0,89-0,98)**
Recent travel	953	0,42	(0,11-1,0)	1,0	(1,0-1,1)
Viral disease n = 708					
**Predictors variables**	**n**	**aLR+**	**(95% CI)**	**aLR-**	**(95% CI)**
**Chest indrawing**	27	**2,9**	**(1,4-5,4)**	**0,98**	**(0,97-0,99)**
**Hepatomegaly**	45	**2,5**	**(1,5-4,1)**	**0,95**	**(0,92-0,98)**
**Runny nose**	464	**2,1**	**(1,8-2,6)**	**0,63**	**(0,59-0,68)**
**Cough**	458	**1,7**	**(1,5-2,0)**	**0,71**	**(0,66-0,76)**
**Fever duration ≤3 days**	888	**1,1**	**(1,1-1,2)**	**0,54**	**(0,42-0,70)**
**Age <5 years**	941	**1,0**	**(1,0-1,1)**	**0,60**	**(0,44-0,80)**
**≥4 liquid stools/day**	45	**0,45**	**(0,26-0,78)**	**1,0**	**(1,0-1,1)**
**Temperature ≥40°C**	37	**0,36**	**(0,20-0,63)**	**1,0**	**(1,0-1,1)**
Seizure	16	0,35	(0,12-1,0)	1,0	(1,0-1,0)
**Jaundice**	42	**0,28**	**(0,16-0,41)**	**1,1**	**(1,0-1,1)**
**Splenomegaly**	44	**0,28**	**(0,16-0,46)**	**1,1**	**(1,0-1,1)**

The best predictors resulting from the multivariate analyses, defined as the one with the highest aLR >1 and the one with the lowest aLR <1 to respectively rule in (RI) and rule out (RO) each disease, were the following: malaria, RI: temperature ≥40°C (aLR+ 8.4, 95% CI 4.7–15); RO: lymphadenopathy (aLR+ 0.07, 0–0.5); typhoid fever, RI: abdominal tenderness (aLR+ 5.9, 2.5–11); RO: dry season (aLR+ 0.56, 0.25–0.89); urinary tract infection (UTI), RI: pollakiuria (aLR+ 5.4, 0.7–16); RO: age >3 years (aLR+ 0.20, 0–0.50); radiological pneumonia, RI: abdominal tenderness (aLR+ 5.7, 1–15); RO: absence of leukocytosis (aLR- 0.55, 0.33–0.76 acute HHV6 infection, RI: leucopenia (aLR+ 3.1, 1.9–4.7); RO: dehydration (aLR+ 0.18, 0–0.75); any type of bacterial disease, RI: chest indrawing (aLR+ 19, 8.2–60); RO: recent travel (aLR+ 0.42, 0.11–1.0); any type of viral disease, RI: chest indrawing (aLR+ 2.9, 1.4–5.4); RO: splenomegaly (aLR+ 0.28, 0.16–0.46). A few other particularly clinically relevant and useful predictors were also found by the multivariate analysis: malaria could be ruled in by recent travel (aLR+ 3.5, 1.4–7.1), typhoid by jaundice (aLR+ 2.5, 1.6–3.6), radiological pneumonia by very fast breathing (respiratory rate ≥ 60 if < 12months; ≥50 if ≥ 12 months old) (aLR+ 3.6, 2.3–5.1) and UTI by fever duration of ≥4 days (aLR+ 1.9, 1.1–2.9). All other predictors found by the multivariate analysis are presented in Table 3.

Some additional significant predictors were found through the bivariate analysis (Table 2), that could be useful to either rule in or rule out the diagnosis in clinical practice even if they were not retained by the multivariate analysis (the LR observed by the clinician while examining patients is indeed the crude rather than the adjusted one): malaria: pale nails (LR+ 4.0, 1.8–9.1), abdominal tenderness (LR+ 2.3, 1.0–5.2); typhoid: severe anemia (LR+ 4.4, 1.0–19), splenomegaly (LR+ 4.1, 1.9–9.2), hepatomegaly (LR+ 4, 1.8–8.9), pale nails (LR+ 3.6, 1.1–11), age ≥5 years (LR+ 3.2, 1.6–6.6) and fever duration of ≥4 days (LR+ 2.1, 1.0–4.1); bacterial disease: dyspnea (LR+ 4.3, 1.8–10), chronic condition (LR+ 3.0, 1.8–4.9), splenomegaly (LR+ 3.0, 1.7–5.3), hepatomegaly (LR+ 2.8, 1.6–5.0) and very fast breathing (LR+ 2.3, 1.5–3.7); viral disease: very fast breathing (LR+ 2.8, 1.4–5.5), abnormal chest auscultation (LR+ 2.6, 1.5–4.4), pale nails (LR+ 0.33, 0.15–0.72), difficulty to eat (LR+ 0.38, 0.21–0.70) and low weight (LR+ 0.45, 0.22–0.92).

**Fig 1:**
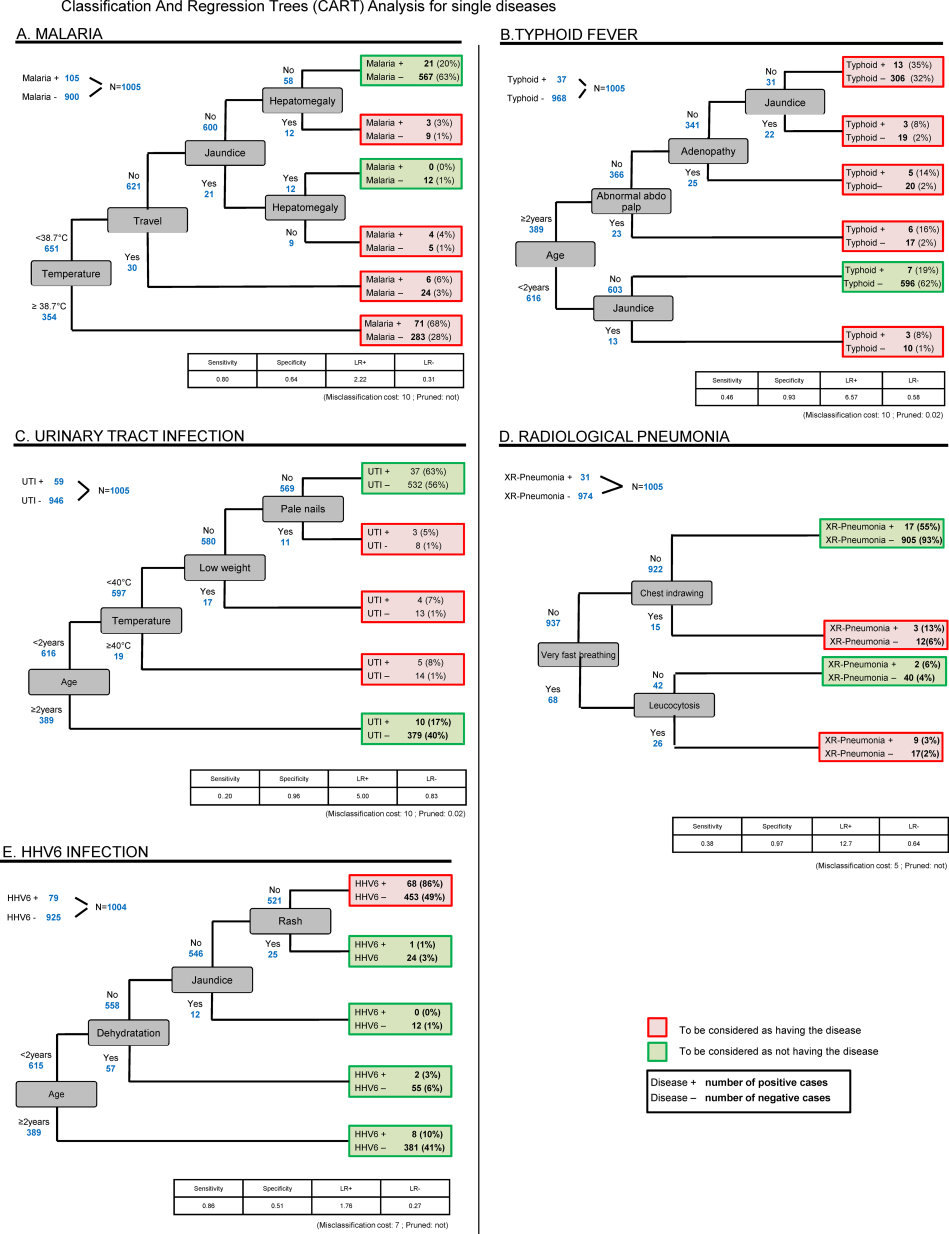
CART analyses for single diseases. Classification and regression trees analyses for single diseases. A. Malaria; B. Typhoid fever; C.Urinary tract infection; D. Radiological pneumonia; E.Human Herpes Virus 6 (HHV6) infection.

**Fig 2:**
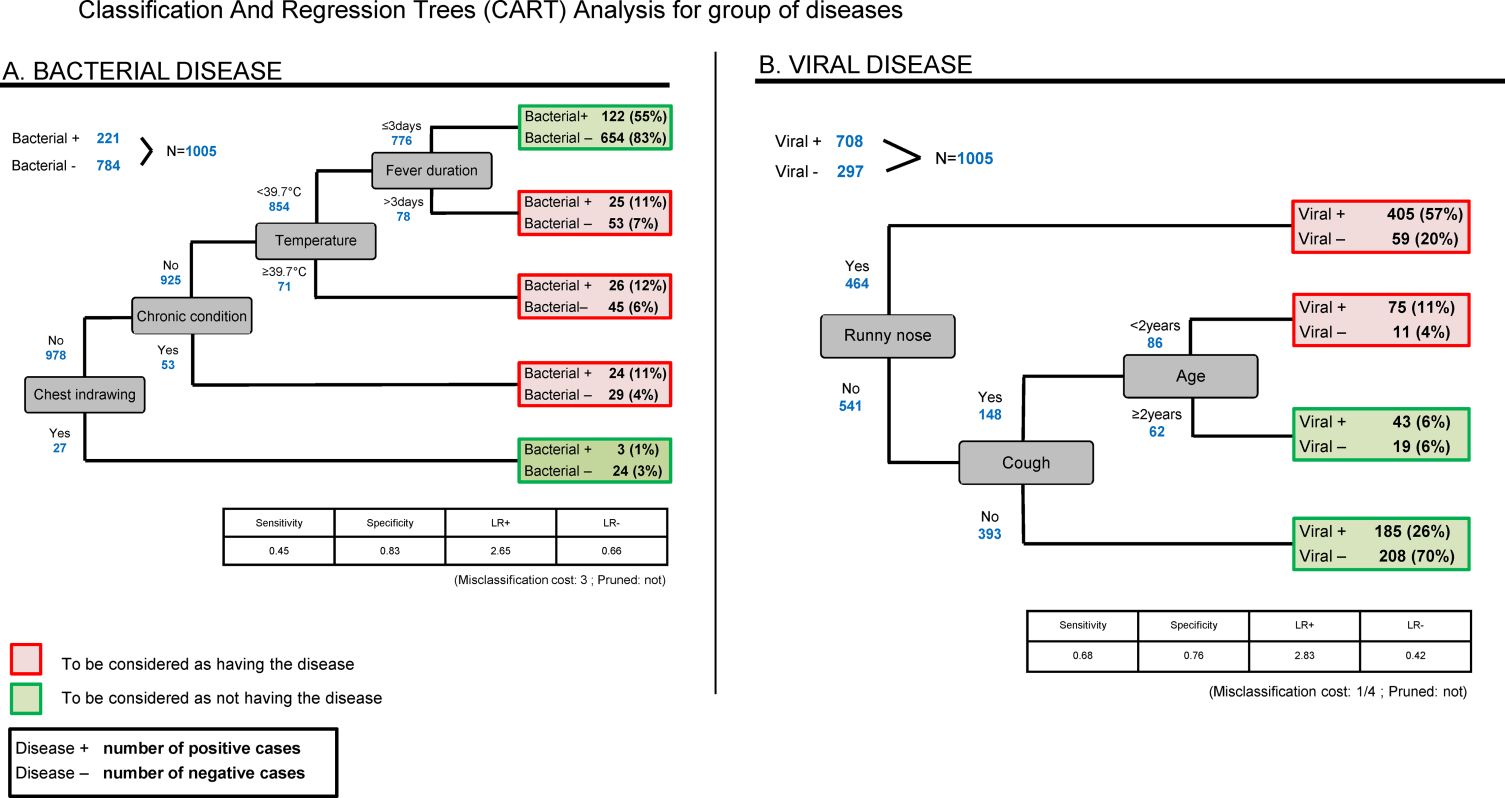
CART analyses for groups of diseases. Classification and regression trees analyses for groups of diseases. A. Bacterial disease; B. Viral disease.

The CART analyses (Figs 1 and 2) show which sequences of clinical parameters best predict the diseases in the format of decision trees. The models providing the best balance between sensitivity and specificity or positive and negative LRs were chosen, provided they made sense from a clinical point of view. Details of parameters introduced in each model are available in S2 Table. For example, a model for malaria using 4 parameters (temperature, travel, jaundice and hepatomegaly) gave a sensitivity of 80%, a specificity of 64%, a LR+ of 2.22 and a LR- of 0.31 and a model for typhoid using 4 parameters (more than 2 years old, jaundice, abdominal tenderness and adenopathy) had a sensitivity of 46%, a specificity of 93%, a LR+ of 6.57 and a LR- of 0.58. The models for the other diseases are presented in Figs 1 and 2.

## Discussion

The present study provides for the first time a list of useful predictors of the main causes of childhood febrile illnesses assessed in an homogeneous population of consecutive patients. The large sample size allowed investigating predictors of several diseases in a real-life cohort of febrile patients, rather than in very well-characterized selected patients.

The great interest of presenting results in LR instead of in odds ratios is that they are independent of the prevalence of the disease in the population (pre-test probability) and can be used directly to calculate the post-test probability using the Fagan’s nomogram [27,28] and integrated in decision making algorithms. The trees generated by the CART models cannot be directly used as such and have to be included in an integrated algorithm that then needs to be prospectively validated. They offer however the great interest of presenting results in a more intelligible way, the possibility to enter continuous variables given that the analysis will select the best cut-off, and the advantage of evaluating the combined accuracy of predictors.

### Malaria

Most predictors of malaria obtained in our analyses (Table 3 and Fig 1A) are consistent with the findings of other studies [2,11,12,29–33]. This gives credit to the predictors we identified for diseases that have been less studied than malaria. High temperature, recent travel and jaundice were retrieved by two different types of analyses (logistic regression and CART). High temperature had the highest LR and was used as the tree’s first node in the CART model; in the latter, 38.7°C was calculated as the best cutoff for temperature to predict malaria. In a systematic review on clinical prediction rules for malaria, fever >37.7°C or >38°C were found as predictive of the disease [11]. Other studies showed an association of high temperature >40°C with severe malaria [13]. No study using CART analyses was found to be able to confirm the threshold for temperature that we found. Recent travel can be explained by the fact that malaria transmission is higher outside than in the towns of Dar es Salaam and Ifakara, and that children use less protective measures, such as impregnated bed nets, during their stay in the rural area than at home. Splenomegaly, which was the second strongest predictor according to the logistic model, does not appear in the CART model and contrariwise, hepatomegaly, which was selected by the chosen CART model, has not been retained by the logistic model. For the latter, a significant interaction with jaundice prevented it from being selected in the logistic model, which explains why these two predictors were mutually exclusive in the different generated CART models.

### Typhoid fever

In the present study, 73% of typhoid fevers occurred in children older than 2 years and the variable age, with a cutoff of 2 years, was in fact used for the first node in the CART model (Fig 1B). Other studies have also shown this association with age [14,16,17]. Age is an easy parameter to assess for clinicians and could be used in guidelines to decide on performing a laboratory test or giving presumptive treatment for typhoid fever. Jaundice, abdominal tenderness and lymphadenopathy were predictive of typhoid in both types of analyses. Jaundice was present in 22% of the typhoid cases, which is a higher prevalence compared to other studies (10 to 16%) [34,35]. However, this sign is not described in most studies as a clinical predictor of typhoid fever in children [14–17,36–39]. This could be explained by the difficulty for many clinicians to assess this sign combined with its relatively low frequency in children in general. Typhoid fever should thus be included in the differential diagnosis of jaundice, besides malaria and hepatitis. Abdominal tenderness was the strongest predictor resulting from the logistic regression and, combined with age in the CART model, these two predictors identified only 17 false positive cases, providing a high specificity of 98%. Abdominal signs in general are well described in the literature as predictors of typhoid fever, and liver pain, which corresponds to an abdominal tenderness in the upper right quadrant, was also a good predictor in our study. Although very specific, this latter sign was however still quite rare among typhoid fever cases (8%), which would preclude its use in clinical guidelines. Rainy season was significantly associated with typhoid fever: 80% of the cases occurred during that season. We did not find any similar result in other studies. The reason might be that during heavy rains, the sewers tend to overflow and contaminate the pipes of clean water with fecal bacteria, including *Salmonella enterica serova Typhi*. Interestingly, 14% of the children with typhoid presented lymphadenopathies. This sign is not clearly described in the literature and further studies would be necessary to really evaluate its clinical predictive value. Prolonged fever, commonly described as a predictor of typhoid fever and one of the reasons for Integrated Management of Childood Ilnesses (IMCI) to recommend referral of children whenever fever lasts more than one week, was significantly associated in the bivariate analysis with a crude LR+ of 2 for fever duration of ≥4 days. In the present study, which included only fevers lasting less than one week, a significant number of typhoid cases were still identified, among which more than a quarter were severe cases based on objective criteria. This means that diagnosing typhoid regardless of the fever duration, either based on clinical predictors such as those found in the present study, or by a reliable test when available, is essential.

### Urinary tract infection (UTI)

In the present study, 95% of children with UTI were under the age of 3 years (the best age cutoff in the bivariate analysis, the CART models (Fig 1C) providing a cutoff of 2 years instead) and 100% under 5 years, which confirms the strong relationship between age and UTI as described in other studies [18,40]. Temperature ≥40°C and fever duration of ≥3 days were also associated with UTI as described in two studies [18,41]. A known symptom of UTI, usually found in school-aged children and adults, pollakiuria, was strongly associated with UTI as well. When present, specific symptoms of UTI are thus of great help to suspect the diagnosis in older children. Considering their good sensitivity, accessibility and frequency, one or more of these parameters could be integrated in guidelines, for example to improve the pre-test probability before performing a urine dipstick. Although the dipstick test has the advantage over urine culture of not being subject to bacterial contamination, it still lacks specificity. To avoid overdiagnosis, this test should thus not be used in all febrile children regardless of the clinical presentation, but rather in a sub-group of children selected on the basis of these predictors. White mouth (oral candidiasis) was associated with UTI as well; both UTI and oral candidiasis most often affect young children and therefore, this predictor probably simply represents a confounding factor.

### Radiological pneumonia

Recent studies conducted in US emergency departments have shown that WHO criteria used to diagnose radiological pneumonia lack sensitivity and specificity [42] and that tachypnea did not discriminate between children with positive and negative chest X-ray [43]. In a systematic review and meta-analysis, fast breathing and lower chest wall indrawing showed poor diagnostic performance [44]. Thus, for settings where radiology is not available, looking for alternative predictors could be useful to improve diagnostic rules. The performance of the widely studied sign tachypnea [19,20,43,45–48], also named fast breathing (WHO terminology), could not be measured in the present study as it was the entry point for deciding to perform a chest X-ray and thus to diagnose radiological pneumonia. However, very fast breathing and other respiratory signs or symptoms, such as dyspnea, chest indrawing, nose flapping and abnormal chest auscultation can still be studied and the bivariate analysis showed that respiratory signs or symptoms presented the highest +LRs. In bivariate and multivariate analyses, the best predictors were respiratory signs, abdominal tenderness, high temperature and leukocytosis. The strongest predictor, abdominal tenderness, which probably reflects the presence of a serious infection in general, should be easy to assess by clinicians and could thus be potentially useful in an algorithm aimed at identifying pneumonia. The predictors abnormal chest auscultation or leukocytosis are clinician dependent for the first and not always available for the second, which makes them unsuited for guidelines. On the contrary, the predictors very fast breathing and temperature ≥39°C are easy to assess. In the CART model (Fig 1D), respiratory signs and leukocytosis were selected but the final sensitivity (0.20) was very low and despite a high specificity (0.97), the performance of the CART is not sufficient. As authors concluded in a recent systematic review on clinical features for diagnosis of pneumonia in outpatients [44], our findings highlight that new point-of-care tests to measure host biomarkers or specific pathogens need to be further investigated to be able to provide better diagnostic tools to clinicians.

### Acute HHV6 infection

With 79 cases, acute HHV6 infection is the second most frequent diagnosis after malaria (105 cases). Only 1% of the children with HHV6 presented a rash, which is described as the most typical feature of this infection (roseola). A US study conducted in 1994 [49] found a prevalence of only 6% of roseola’s typical exanthema at the beginning of the episode and a total of 17% when including exanthema appearing when fever subsided. An explanation could be that the faint rash of acute HHV6 infection is difficult to detect on black skin and generally appears after the resolution of the fever. Both the logistic regression (Table 3) and the CART models (Fig 1E) found that age under 2 years was useful to predict HHV6 infection. Leucopenia was also predictive of HHV6 in accordance with the literature [50]. Interestingly, the CART model shows that it is the absence rather than the presence of each symptom or sign that rules in HHV6. We therefore looked at the association between the variable absence of clinical symptom or sign and HHV6 and found a crude LR+ of 1.8 (1.1–2.8). This finding supports the recommendation provided by the WHO and other guidelines not to treat febrile children with antibiotics in the absence of specific symptoms or signs and to rather follow-up with the patient, as most of these cases correspond to self-limited viral infections.

### Bacterial disease

The predictors found for (any type of) bacterial disease, temperature ≥40°C and seizures, were described in the systematic review of Thompson et al. [51]. We also found chest indrawing and nose flapping, predictors which were not described in other studies. These signs reflect the presence of a respiratory distress that can be due to severe sepsis in general, and not only to a respiratory infection. Severe anemia, that is known to lead to a high mortality rate, is well known in malaria but not specifically as a predictor of bacterial infection. Hemoglobinometer could thus potentially be used as a triage tool at primary care level to detect children at risk of suffering from a serious infection, and thus allow to decide to refer them at a higher level of care with an antimalarial and an antibiotic as pre-referral treatment. The decision tree of the CART model (Fig 2A) used chest indrawing, chronic condition, temperature >40°C and fever duration of >3 days to predict bacterial disease, resulting in an overall good specificity of 0.83, but unfortunately a rather poor sensitivity of 0.45. The aLR+ of the predictors in the logistic regression model were indeed quite high, which allows to rule in the diagnosis, but their aLR- being included between 0.85 and 0.97, their absence does not allow to exclude a bacterial infection. Abdominal tenderness, which was a good predictor of the three infections at highest risk of bad outcome (radiological pneumonia, typhoid and malaria), did not come out for bacterial disease. This is because this category did not include malaria and included also milder bacterial infections, such as UTI or skin infections. Abdominal tenderness thus seems to be a predictor of a serious infection and could potentially be used at primary care level to either refer the child and/or treat him/her with an antibiotic.

### Viral disease

Regarding viral disease, the most interesting predictors were negative predictors. These corresponded to the absence of severity criteria, such as seizure, temperature > 40°C or jaundice, which illustrates that the clinical presentation of viral diseases is more often mild than that of bacterial diseases. However, chest indrawing, a criterion used to classify pneumonia in WHO guidelines, was in fact associated with viral disease in the multivariate regression. This is due to the fact that most acute respiratory infections, especially in young children, are due to viruses rather than bacteria, even in children with a more severe presentation. In the northern countries, bronchiolitis is nowadays one of the main causes of hospital admission in children. With the epidemiological transition due to many factors, including vaccines against pneumococcus and *Haemophilus*, the southern countries are also progressively shifting from bacterial to more viral diseases. To avoid overtreatment of febrile children with antibiotics, a public health priority to mitigate the spread of microbial resistance, it is essential to also provide clinicians with prediction rules able to identify children with viral infections.

## Limitations of the study

The presence in the study of 2% of children with two diagnoses of the five diseases analyzed could have slightly decreased the accuracy of our results. However, excluding these children to get ‘purer’ predictors for each diagnosis would not have reflected the reality of febrile patients attending health facilities who often present more than one infection, or for whom it is very difficult to know which of the several infections documented are the cause of the acute fever. Another limitation is the fact that for two diseases (radiological pneumonia and UTI), the confirmatory test was not performed in all children but rather in a sub-group of patients with a higher pre-test probability. The aim was to avoid overdiagnosis due to the well-known lack of specificity of the available tests, as well as unnecessary testing, in particular X-ray exposure. This impeded us to study some of the variables because they were included in the case definition, and misclassification of disease cannot be excluded. Although the overall sample size was large with 1005 children, the subgroups of patients for some of the diseases were relatively small, which reduced the statistical power. This problem is inherent to the fact that the study included all consecutive patients presenting to the clinic with fever, to avoid the selection bias often found in studies on diagnostic accuracy. Moreover, due to the high number of variable tested for each outcome some of the associations found might represent confounding factors rather than true predictors, reason why a multivariate analysis was also performed to explore this possibility.

## Implications for clinical algorithm development

The present findings provide evidence that can be used to develop new clinical algorithms or improve existing ones. Data were collected in two representative settings of Tanzania in the absence of known outbreaks, which makes their external validity quite strong, at least throughout Tanzania. Some of the predictors evaluated thanks to the logistic regression analysis have already been used to develop an updated version of IMCI called ALgorithm for the MANAagement of CHildhood Ilness (ALMANACH) [52] that has been evaluated in the same patient population (pediatric outpatients in Dar es Salaam and Ifakara). This new algorithm allowed to drastically decrease the use of unnecessary antibiotics (from 84 to 15%) while still improving the clinical outcome of patients (the clinical cure rate at day 7 increased from 92 to 97%) [53]. Additional research is desirable to know whether these results can be extended to settings outside Tanzania and even beyond Sub-Saharan Africa. Because of the constant evolving and changing epidemiological context, similar studies will anyway have to be repeated in the future to update existing algorithms.

The way clinical predictors can be used in algorithms for the management of patients depends on several factors. When a reliable and easy-to-perform test is available, such as for malaria, predictors are useful to decide on which patients to test, for example in low endemic areas where the pre-test probability becomes too low to justify testing all febrile patients. In contrast, for typhoid fever, clinical predictors are useful to diagnose the disease when blood cultures are not available and while waiting for the availability of a sensitive rapid test. In both situations, the aim is to push the post-test probability, either above the threshold for treatment (the diagnosis is included), or below the threshold for further testing (the diagnosis is excluded) [27]. According to these testing and treatment thresholds, predictors with a high LR+ (and reasonable LR-) or rather a low LR- (and reasonable LR+) will be chosen. Another parameter to take into account is the prevalence of the predictor in the affected population: if this predictor is strong but rarely found in patients having the disease, only few patients will be identified and it is not worth integrating it into the algorithm. For typhoid, liver pain, jaundice and abdominal tenderness had the highest LR+ and were present in 8%, 22% and 19% of the children with typhoid. Liver pain can thus be left out (also because it is a difficult sign for clinicians to assess). Using the other two predictors in parallel would allow detecting 32% of children with typhoid (probably the most advanced cases) and falsely diagnose 6% of children without typhoid. Adding another predictor could slightly increase the number of typhoid patients detected, but at the cost of considerably increasing the number of children treated unnecessarily with antibiotics. Predictors can also be combined by using them in series rather than in parallel. This is especially useful when the purpose is to select patients for a diagnostic test in two situations: 1) to avoid overtreatment when the test is sensitive but not specific enough; 2) to save diagnostic tests, when the pre-test probability of disease is low. Diagnosis of UTI using urine dipstick (as urine culture is not available in most African settings and gives a result after several days only) is a good example of the first situation. Age being one of the strongest predictors of UTI and an easy information to retrieve for clinicians, algorithms could include urine testing only for children younger than 2 or 3 years. Malaria testing in very low endemic areas is a good example for the second situation.

The CART models found for UTI and for HHV6, the two most prevalent infections in children aged less than 2 years outside respiratory infections and diarrhea, were both based on the absence of other symptoms and signs. These two infections, that require the opposite attitude regarding antibiotic prescription, are indeed the main causes of non-specific fevers, a clinical situation for which management guidelines are desperately missing. The lack of (and difficulty to develop) a fully reliable and easy-to-use microbiological test for these two infections, implies that other types of biomarkers should be looked for, for example markers of the host reaction. Interestingly, in our study, leucopenia was a good predictor allowing to both rule out UTI and rule in HHV6, and could thus be potentially used in young children presenting non-specific fevers and enable clinicians to decide whether antibiotics should be prescribed or not. It would certainly be worth doing a clinical trial to assess the usefulness of leucopenia, especially now that point-of-care tests able to measure white cell counts are coming on the market. In the bivariate analysis, another interesting parameter, fever duration of more than 4 days, was found to be predictive of two important bacterial infections that are challenging from the diagnostic point-of view as they often present clinically as fevers without focus: UTI and typhoid fever. The reason might be that these two diseases tend to get progressively worse over time, which motivates the caregivers to consult only after several days of fever, while in contrast viral respiratory diseases tend to present abrupt marked symptoms but that improve rapidly. This finding supports the WHO’s recommendation to refer children with prolonged fever (more than 7 days) to a higher level of care for further investigations. However, the cutoff of 7 days chosen by WHO is too long and implies that many cases, presenting at a moment where antibiotics would probably already be beneficial, are missed.

Finally, even if the combinations of predictors that we found for bacterial disease and for viral disease in the CART analyses had limited accuracy, they included interesting predictors, such as the presence of a chronic condition, very high temperature or age. These parameters are easy to assess and might increase the post-test probability if included in new decision-making rules and algorithms, after having been tested in clinical trials to find out the best combinations that should be used.

## Conclusion

This study, which included consecutive children presenting with fever in outpatient clinics, allowed to generate objective information on predictors, based on the real prevalence of clinical parameters and diseases with direct comparison between the main diseases causing fever. Some predictors were similar to those found in previous studies including patients with one specific disease only, and others were new. The predictors discovered in this study can be used to create new clinical decision rules and to update practice guidelines for the management of febrile children. In addition, the CART analyses, which allow to combine several clinical predictors in the best way and in a format of a decision tree is an important addition to the conventional logistic regression analysis. Several parameters demonstrated an association with the outcome in both analyses, which increases their robustness.

In many parts of the world, clinical predictors are often the only tools that health workers have in hand to make a probabilistic diagnosis and decide on an appropriate treatment for a fever episode. This study, as many others, demonstrates the overall lack of sensitivity and specificity of prediction rules based only on symptoms and signs, and the need to complement the approach with point-of-care laboratory tests. It is still crucial that health workers know about the relative performance of these predictors to be able to better decide when to test, treat, refer or simply observe a child, with the final aim being to decrease morbidity and mortality, but also to avoid unnecessary antimicrobial prescription.

## Supporting information

S1 Table Variables not predictive of any of the diseases investigated. (DOCX)

S2 Table CART models. The selected models showed in Figs 1 and 2 are highlighted in yellow.
(PDF)
