# Supplementary material for: Predictive value of clinical and laboratory features for the main febrile diseases in children living in Tanzania: A prospective observational study
Source: PLoS One. 2017 May 2;12(5):e0173314. doi: 10.1371/journal.pone.0173314 (PMC5413055; doi:10.1371/journal.pone.0173314)
Supplement: S1 Table — (DOCX) [file pone.0173314.s001.docx]

**S1 Table 1.** **Variables not predictive of any of the diseases investigated.**

| Someone sick in the environment |
| --- |
| Rural versus urban site |
| Fever duration of ≤ 1 day |
| Fever duration of ≤ 2 days |
| Cough duration of ≤ 3 days |
| Cough duration of > 3 days |
| Vomiting |
| Diarrhea |
| Abdominal pain |
| Urinary pain |
| Earache |
| Red tympanum |
| Bulging tympanum |
| Tonsillar hypertrophy |
| Mouth ulcer |
| Fever as only symptom |
