# Supplementary material for: Predictive value of clinical and laboratory features for the main febrile diseases in children living in Tanzania: A prospective observational study
Source: PLoS One. 2017 May 2;12(5):e0173314. doi: 10.1371/journal.pone.0173314 (PMC5413055; doi:10.1371/journal.pone.0173314)
Supplement: S2 Table — The selected models showed in Figs 1 and 2 are highlighted in yellow. (PDF) [file pone.0173314.s002.pdf]

Model	Variables included (in factor or continuous)	Variables exclued	Missclassification cost	Pruned	Minsplit	Maxdepth	Sensitivity	Specificity
malaria1	malaria,"agegroup2b","travel","convul","headache","coughd"(continu),"breath","diarrhea","urinefreq","rash","tempa"(continu),"chestindra","chestausc01","jaundice","palenails","adenopath","tympbulg","abdopalp01","liverbig","liverpain","spleenbig"	anemia, severe anemia, thrombopenia, no leucocytosis, leucopenia, no lymphocytosis, hightemp, veryhightemp, cough3d,	10	not	10	4	0.8	0.64
malaria2	malaria,"age"(en continu),"travel","convul","headache","coughd"(continu),"breath","diarrhea","urinefreq","rash","tempa"(continu),"chestindra","chestausc01","jaundice","palenails","adenopath","tympbulg","abdopalp01","liverbig","liverpain","spleenbig"	agegroup2b, anemia, severe anemia, thrombopenia, no leucocytosis, leucopenia, no lymphocytosis, hightemp, veryhightemp, cough3d,	10	not	10	4	0.76	0.73
malaria3	malaria,"agegroup2","travel","convul","headache","coughd"(continu),"breath","diarrhea","urinefreq","rash","tempa"(continu),"chestindra","chestausc01","jaundice","palenails","adenopath","tympbulg","abdopalp01","liverbig","liverpain","spleenbig"	agegroup2b, age en continu, anemia, severe anemia, thrombopenia, no leucocytosis, leucopenia, no lymphocytosis, hightemp, veryhightemp, cough3d,	10	not	10	4	0.76	0.76
malaria4	idem malaria 1	idem malaria1	5	not	10	4	0.43	0.93
malaria5	idem malaria 1	idem malaria1	5	0.022	10	4	0.34	0.97
malaria6	idem malaria 1	idem malaria1	7	not	10	4	0.8	0.64
malaria7	idem malaria 1	idem malaria1	7	0.015	10	4	0.8	0.64
malaria8	idem malaria 1	idem malaria1	12	not	10	4	0.73	0.65
malaria9	idem malaria 1	idem malaria1	15	not	10	4	0.73	0.66
malaria10	idem malaria 1	idem malaria1	15	0.037	10	4	0.73	0.66
malaria11	idem malaria 1	idem malaria1	8	not	10	4	0.8	0.64
malaria12	idem malaria 1	idem malaria1	9	not	10	4	0.8	0.64
malaria13	idem malaria 1	idem malaria1	9	0.021	10	4	0.77	0.64
typhoid1	typhoid,"age"(continu),"season01","diffdrink","feverd"(continu),"tempa"(continu),"dehydrat","jaundice","palenails","mouthulcer","adenopath","abdopalp01","liverbig","liverpain","spleenbig"	severe anemia, highALT, agegroup2b (5 ans), feverm3d, veryhightemp	10	not	10	4	0.54	0.92
typhoid2	typhoid,"age"(continu),"season01","diffdrink","feverd"(continu),"tempa"(continu),"dehydrat","jaundice","palenails","mouthulcer","adenopath","abdopalp01","liverbig","liverpain","spleenbig"	severe anemia, highALT, agegroup2b (5 ans), feverm3d, veryhightemp	10	0.03	10	4	0.38	0.95
typhoid3	typhoid,"agegroup2(cutoff 2years),"season01","diffdrink","feverd"(continu),"tempa"(continu),"dehydrat","jaundice","palenails","mouthulcer","adenopath","abdopalp01","liverbig","liverpain","spleenbig"	severe anemia, highALT, agegroup2b (5 ans), age(continu), feverm3d, veryhightemp	10	not	10	4	0.49	0.94
typhoid4	idem typhoid3	idem tyhpoid3	10	not	10	3	0.4	0.95
typhoid5	idem typhoid3	idem tyhpoid3	15	not	10	3	0.38	0.96
typhoid6	idem typhoid3	idem tyhpoid3	20	not	10	3	0.81	0.62
typhoid7	idem typhoid3	idem tyhpoid3	20	not	10	4	0.46	0.94
typhoid8	typhoid,"agegroup2(cutoff 2years),"season01","diffdrink","feverm3d","tempa"(continu), "dehydrat","jaundice","palenails","mouthulcer","adenopath", "abdopalp01","liverbig","liverpain","spleenbig"	severe anemia, highALT, agegroup2b (5 ans), feverd, veryhightemp	20	not	10	4	0.46	0.93
typhoid9	idem typhoid8	idem typhoid8	10	not	10	4	0.49	0.94
typhoid10	idem typhoid8	idem typhoid8	10	0.02	10	4	0.46	0.93
typhoid11	idem typhoid8	idem typhoid8	10	not	10	3	0.41	0.95
typhoid12	idem typhoid8	idem typhoid8	15	not	10	3	0.38	0.96
typhoid13	idem typhoid8	idem typhoid8	20	not	10	3	0.81	0.62
typhoid14	idem typhoid8	idem typhoid8	12	not	10	4	0.46	0.95
typhoid15	idem typhoid8	idem typhoid8	12	0.03	10	4	0.46	0.93
typhoid16	idem typhoid8	idem typhoid8	5	not	10	4	0.43	0.95
typhoid17	idem typhoid8	idem typhoid8	10	0.02	10	6	0.46	0.93
UTI1	uti,"agegroup2","feverm3d","urinefreq","weightlow","veryhightemp","palenails","mouthwhite","liverbig","liverpain", "leucopenia","lymphocytosis"	none	10	0.02	10	4	0.2	0.97
UTI2	uti,"agegroup2","feverm3d","urinefreq","weightlow","veryhightemp","palenails","mouthwhite","liverbig","liverpain", "leucopenia","lymphocytosis"	none	10	not	10	4	0.23	0.98

radiological pneumonia	pneumoniadoc,"chronicsorted", "convul","breath", "dehydrat", "urinefreq", "weightlow","hightemp", "tachycardia", "veryfastbreath", "chestindra", "noseflap" ,"mouthwhite","adenopath","abdopalp01", "liverpain", "thrombopenia", "leucocytosis", "lymphopenia"	none	5	not	10	4	0.38	0.64
hhv6 1	"hhv601","age"(continu),"travel","diffdrink","headache","rash","earpain","tempa"(continu),"dehydrat","jaundice","mouthwhite"	leucocytosis, leucopenia, lymphopenia, highALT, agegroup2, childmorethree, agegroup2b, veryhightemp	5	not	10	4		
hhv6 2	"hhv601","agegroup2","travel","diffdrink","headache","rash", "earpain","tempa"(continu),"dehydrat","jaundice","mouthwhite"	leucocytosis, leucopenia, lymphopenia, highALT, age(continu), childmorethree, agegroup2b, veryhightemp	5	not	10	4	0.14	0.98
hhv6 3	idem hhv62	idem hhv62	10	not	10	4	0.87	0.47
hhv6 4	idem hhv62	idem que hhv62	7	not	10	4	0.13	0.98
hhv6 5	idem hhv62	idem hhv62	9	not	10	4	0.86	0.5
hhv6 6	idem hhv62 with agegroup2b (cutoff 5ans)	idem hhv62	5	not	10	4		
hhv6 7	idem hhv62 with agegroup2b (cutoff 5ans)	idem hhv62	10	not	10	4	0.15	0.96
hhv6 8	idem hhv62 with childmorethree	idem hhv62	10	not	10	4	0.9	0.33
hhv6 9	idem hhv62 with childlessone	idem hhv62	5	not	10	4	0.54	0.84
hhv6 10	idem hhv62 with childlessone	idem hhv62	10	not	10	4	0.67	0.75
hhv6 11	idem with hhv62 with childlessone	idem hhv62	7	not	10	4	0.66	0.77
hhv6 12	idem with childlessone and hightemp	idem hhv62	5	not	10	4	0.22	0.95
hhv6 13	idem with childlessone and hightemp	idem hhv62	10	not	10	4	0.67	0.75
hhv6 14	idem with childlessone and veryhightemp		5		10	4	0.67	0.75
hhv6 15	"hhv601","agegroup2","travel","diffdrink","headache","rash", "earpain","veryhightemp","dehydrat","jaundice","mouthwhite"	labo and continuous (age and tempa)	7		10	4	0.86	0.51
bactdisease 1	"bactdisease","travel","chronicsorted","feverm3d","convul","breath","rash","weightlow","tempa","veryfastbreath","chestindra","noseflap","jaundice","palenails","mouthwhite","adenopath","tympbulg","liverbig","liverpain","spleenbig"	severanemia, feverd, veryhightemp	5	not	10	4	0.45	0.83
bactdisease 2	idem bactdisease1	idem bactdisease1	2	not	10	4	0.37	0.89
bactdisease 3	idem bactdisease1	idem bactdisease1	10	not	10	4	1	0.04
bactdisease 4	idem bactdisease1	idem bactdisease1	3	not	10	4	0.45	0.83
viraldisease 1	"viraldisease","age"(continue),"diffeat","feverm3d","convul", "runnose","cough","coughd"(continu),"diarrheam3n","earpain","weightlow","tempa"(continu),"fastbreath","veryfastbreath","chestindra","noseflap","chestausc01","jaundice","palenails","mouthwhite","liverbig","liverpain","spleenbig"	agegroup2b (5ans), feverd, coughm3d, cough3d, veryhightemp	2	not	10	4	0.99	0.17
viraldisease 2	idem	idem	1	not	10	4	0.93	0.36
viraldisease 3	"viraldisease","agegroup2b","agegroup2","childmorethree","childlessone","diffeat","feverm3d","convul","runnose","cough","coughm3d","diarrheam3n","earpain","weightlow","tempa","fastbreath","veryfastbreath","chestindra","noseflap","chestausc01","jaundice","palenails","mouthwhite","liverbig","liverpain","spleenbig"	age, feverd, coughd, veryhightemp	1	not	10	4	0.99	0.17
viraldisease 4	idem	idem	10	not	10	4	0.19	0.97
viraldisease 5	idem	idem	4	not	10	4	0.68	0.76
